# Throat Ail

**Published:** 1884-01

**Authors:** 


### THROAT AIL.

THE first most frequent cause of this malady, in our opinion, is indigestion, the lining of the throat being a continuation of the lining of the stomach—one source of nerve-supply to both throat and stomach, being also identical.

There is reason to believe that the second most frequent origin of this troublesome, mischievous and dangerous ailment, is the irregular and injudicious exercise of the vocal organs on the part of singers, clergymen, and other speakers.

The muscles of the human body are under the same general physical laws; they are all developed and strengthened by moderate, regular exercise. If a man runs a hundred yards to-day it will tire him—if he do it a week hence the same result will be observed, for successive months. But if he runs a hundred yards to-day, and repeats it day after day, we all know that, if in ordinary health, the ease with which that race can be performed will become greater and greater, until it can be done without a scarcely appreciable effort. And so it would assuredly be if clergymen would speak in public, in moderation, every day, and when this is not practicable, I have advised the daily reading out aloud in a conversational tone. with the same animation usual to the delivery of a sermon.

The non-use or the spasmodic exercise of any muscle in the human body, will result injuriously, if persevered in.

Another item is of important consideration, and it is a point, too, on which every public speaker should keep his eye steadily fixed. It is not the irregular exercise of the vocal organs, of itself, which originates most of the difficulty; it is that such exercise leaves the throat in a heated and debilitated condition, and in that condition, being suddenly exposed to an altered temperature, injury is the result, on principles precisely the same as are in operation under circumstances of checked perspiration. Single injuries of such a nature would be comparatively harmless, but when repeated weekly for successive years, it is not strange that troublesome symptoms are a permanent result.

There are two forms of throat-ail—the rapid and the slow. By rapid throat-ail, the great and good Washington perished prematurely, in a few hours' illness. By the slow kind, many public men are deprived of their means of usefulness, and of support.

*Throat-Ail* is like a fire, the sooner you know of its existence the better; and like a fire too which seldom goes out of itself; so throat-ail seldom indeed gets well of itself, but burrows and deepens, until it undermines the constitution, wastes away the health, and strength, and flesh, and finally fastening itself in the lungs, completes the wreck and ruin of the whole man.

The first symptoms of Throat-Ail, or Chronic Laryngitis, or Clergymen's Sore Throat, are usually a frequent hemming and hacking, in order to clear the voice or throat; this is slight and seldom at first, and may not be noticed for weeks; but then, it is so decided, that it forces itself upon the attention, either by its frequency, or by the force required to clear the throat sufficiently to speak with distinctness. After a while, it requires such an effort to enunciate plainly, that the patient for the first time becomes aware of a certain feeling of tiredness about the throat or neck; most generally it is a dull hurting; or he finds there is a kind of lumpish feeling in the throat, and he attempts to swallow it away, and it does seem to go down, but it does not stay down, and he swallows again, and soon he finds himself *swallowing all the time;* occasionally there is a different cause for swallowing, the throat appears to be dry, and swallowing for a time seems to moisten it; finally the swallowing is almost incessant, especially if the mind is directed to it. For a time, nothing is brought away; gradually a little pearly or whitish or cottony like phlegm is brought up, and the patient becomes hoarse. In the progress of things this phlegm becomes dryish, and so tough, that it clings to the inside of the throat, and can only be dislodged by a decided effort at clearing, with a dipping forward of the head. The voice next becomes husky; at last a positive cough is necessary to dislodge the phlegm, and consumption soon follows.

The symptoms detailed are present in the history of every case I have known. Accompanying these, there are occasional additional symptoms. A kind of pain, sharp or hurting, runs up the side of the neck towards the ear. Some complain of a burning feeling now and then at the little hollow at the bottom of the neck; or up and down the breast bone in the centre, or at the pit of the stomach. These burning sensations are not felt continuously in any case, but at certain times during the day.

A very common symptom is a depression of spirits, altogether greater than the actual feeling of discomfort warrants. In the progress of the disease, the feet bocome cold; there is a bad taste in the mouth of mornings; occasional headache; the bowels do not act daily, or if they do, what is passed is hard or bally; the patient is easily chilled; "the slightest thing in the world" gives him a cold, and "a cold always makes the throat worse." The food either sours on the stomach, or remains there like a weight for hours at a time; the appetite becomes impaired, or it is so voracious, that "I can eat almost any thing" and "yet hungry all the time." The patient begins to lose flesh and strength; and does not swallow as easily as he used to; at length he cannot swallow at all; in the effort, even water comes back through the nose and the man dies of starvation.

*Three cases out of every four coming to me for throat-ail, have it as the result of improper eating and drinking.*

Such a large proportion of cases of throat-ail originating in the stomach, I found my remaining remarks on this general origin.

